# Supplementary material for: Atypical lymphocytes in the peripheral blood of COVID-19 patients: A prognostic factor for the clinical course of COVID-19
Source: PLoS One. 2021 Nov 12;16(11):e0259910. doi: 10.1371/journal.pone.0259910 (PMC8589156; doi:10.1371/journal.pone.0259910)
Supplement: Attachment — Submitted filename: Responce to Reviewers.doc [file pone.0259910.s003.doc]

October 24 2021

Dr. Baochuan Lin,

Academic Editor,

PLOS ONE

Re: Manuscript ID: PONE-D-21-21070R1

Dear Dr. Baochuan Lin,

Thank you very much for your e-mail and review of the manuscript (PONE-D-21-21070R1) that we sent on September 14, 2021. We thank you and the two reviewers for providing constructive comments regarding the improvement of the original manuscript.

Here, we are sending a PDF file of our revised manuscript. All changes have been made in response to your suggestions, and itemized responses to the individual reviewer’s comments are also attached.

Response to comments:

1. Line 147 - 148: The sentence "There were 9 patients with 1% atypical lymphocytes" was repeated, please delete one.

Response: We thank the editor for kind advice. The duplicative sentence was deleted as indicated.

2. Line 282 - 283: "This may be thought to prove otherwise." Not sure what the authors wish to convey, please rephrase for clarification.

Response: We thank the editor for pointing out an insufficient sentence in our manuscript. We rephrased this sentence as " This may be thought to prove that the activation of T cells was not related to recovery from COVID-19".

3. Line 284: Should "Thought" be "Though"?

Response: Yes. We thank the editor for kind advice, and correct the word as indicated.

4. Line 286: Suggest changing "seemed" to "was".

Response: We thank the editor for this helpful suggestion, and correct the sentence as indicated.

5. Line 296: Suggest changing "occasionally" to "sometimes"

Response: We thank the editor for this helpful suggestion, and correct the sentence as indicated.

6. Line 304: Delete "to"

Response: We thank the editor for kind advice. The sentence was corrected as indicated.

We believe that we have addressed the comments from the academic editor and both reviewers. We look forward to hearing from you regarding our submission. We would be glad to respond to any further questions and comments that you may have. Thank you for your generous consideration.

Sincerely yours,

Jun Sugihara, MD

Department of Respiratory Medicine, Tokyo Medical and Dental University, 1-5-45 Yushima, Bunkyo-ku, Tokyo, 113-8510, Japan

Phone:	81-3-5803-5954

Fax:	81-3-5803-0260

Email:	sugihara.pulm@tmd.ac.jp
